# Reducing translational science roadblocks to disability research

**DOI:** 10.1017/cts.2025.10073

**Published:** 2025-06-09

**Authors:** Karen Bonuck, Ariel Fishman

**Affiliations:** 1 Departments of Family and Social Medicine, and Pediatrics Co-Director, University Center of Excellence in Developmental Disabilities, Institute for Clinical and Translational Research, Albert Einstein College of Medicine- Montefiore Medical Center, Bronx, NY, USA; 2 Department of Epidemiology and Population Health, Institute for Clinical and Translational Research, Albert Einstein College of Medicine-Montefiore Medical Center, Bronx, NY, USA

**Keywords:** Disability, National Institutes of Health designation, health disparity population, Clinical and Translational Science Awards, developmental disability

## Abstract

Disability is a common and universal human experience. Yet, people with disabilities (PWDs) are in poorer health and have less access to quality healthcare than their non-disabled peers. In fact, the National Institutes of Health’s (NIH) designated PWDs as a health disparities population in 2023. This paper illustrates the application of translational science (TS) principles to overcoming roadblocks to reducing PWDs’ health disparities. Part I provides an overview of health disparities among PWDs and the recent designation – situating both within a TS framework. Part II summarizes literature on specific factors that contribute to PWDs’ exclusion from research, how these factors are reflected in background reports that impelled the designation of PWDs as a disparity population, and how the suggested steps to implement the designation reflect TS principles and its research agenda. Part III describes “Reducing Researcher Roadblocks to Including People with Disabilities in Research (D2/R3),” a TS solution to overcoming PWDs exclusion from research. D2/R3 is our institution’s Clinical and Translational Science Award research project – a mixed-methods study that targets research teams’ knowledge, attitudes, biases, and perceptions that contribution to under representation of persons with developmental disabilities in research.

## Introduction

Terminology: DDs refer to broad disabilities that can be intellectual, physical, or both. “IDD” is the term used when both an intellectual disability (ID) and another disability are present. ID encompasses intellectual functioning and adaptive behavior differences. IDDs begin in childhood (<22 years), impact physical, cognitive, and/or social function, and are often lifelong. They include ADHD, autism, cerebral palsy, learning disability, deafness, and blindness. NB: the term “impairment” refers to physiological or functional attributes, while “disability” refers to societal barriers that prevent people with impairments from full participation.

## Disability as a health disparity in the context of translational science

### Disability health disparities

About one in four US adults identify as having a disability or impairment affecting their mobility, cognition, independent living, hearing, vision, and/or self-care [1]. Disability may present as salient to others or be non-visible (e.g., autism, Long COVID). The National Advisory Council to the National Institute of Minority Health and Health Disparities Working Group on Persons Living with Disabilities found “robust evidence” of poorer health, greater disease burden, and lower access to quality health care among persons with disabilities (PWDs) [2]. Adults with (vs. without) disabilities have higher rates of obesity (41.6% vs. 29.6%), smoking (21.9% vs. 10.9%), heart disease (9.6% vs. 3.4%), and diabetes (15.9% vs. 7.6%), and lower rates of preventive care (e.g., mammograms, cervical cancer screening) [3]. Yet, PWDs remain under-represented in clinical research about conditions separate from those caused by their disability or its functional impact.

**Figure 1.**
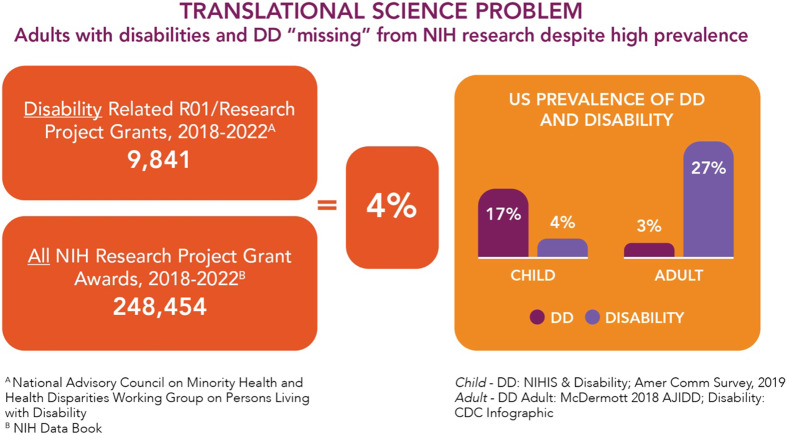
US disability research vs. Prevalence of developmental disability (DD) and all disability, by age. Legend: NHS = national.

Though 25% of adults identify as a PWD, there is limited research on their health. Between 2018–2022, the NIH funded nearly 250,000 research grants, of which 10,000 were disability related – representing just 4% of its portfolio (Figure 1). Driven by this imbalance, PWDs were designated a population experiencing health disparities for NIH research. The announcement of the designation noted that PWDs experience a wide range of health conditions that lead to poorer health and shorter lifespan along with “…discrimination, inequality, and exclusionary structural practices, programs, and policies [that] create barriers to timely and comprehensive health care.” People with disabilities who also belong to one or more other populations with health disparities fare even worse [4].” The designation drew from a report [5] recommending that the NIH (a) establish an Office of Disability Research; (b) issue funding announcements regarding PWDs who have co-occurring chronic conditions and/or who are members of other health disparity populations; and (c) institutes and centers conduct portfolio analyses to identify gaps in research of certain disabling conditions and issue funding opportunities for them. As a discipline that studies the structure and process of scientific work, translational science (TS) principles are reflected in this recent designation. Indeed, as discussed in Part II, much of the work surrounding the designation embodies TS approaches, even if not explicitly framed as such.

### Translational science and the culture of science

TS encompasses changes in the structure and process of scientific work (discussed in Part II) and in the culture of science, including its values, assumptions, and meanings [6]. The *culture of science* regards disability as an outcome to prevent or treat, eschewing maximization of health and equity for people who already have disabilities [7]. Reflecting momentum to reshape the culture of science away from a purely “medical model,” the NIH designation included a recommendation to replace its use of the terminology “reducing disability” with: “…to enhance health, lengthen life, and reduce illness.” Such change aligns with disability advocates move away from a “medical model” of disability as an individual defect to be treated, towards a “social model” that views disability as arising from social and environmental barriers independent of impairments. In the medical model, a person with a spinal cord injury cannot ascend a staircase because their injury affects use of their legs; under the social model, their ability to ascend is impeded by the lack of a wheelchair-accessible elevator. In addition. community-based participatory research methods that meaningfully engage PWDs could support these changes in the culture of science and is consistent with the disability rights tenet of “nothing about us without us [8].” Such engagement includes outreach to the disability community during the conceptualization phase so that research questions reflect PWDs lived experiences, inclusion of PWDs on the research team, and timely dissemination of results in accessible formats.

TS is uniquely poised to catalyze the culture of science in ways that advance the NIH designation goal of reducing health disparities of PWDs. As described in Part II, the major factors associated with PWDs exclusion from research can be mapped to both recommendations from an NIH Director’s Subgroup on Individuals with Disabilities (hereafter “subgroup”) [9], and TS principles. For example, overcoming eligibility and recruitment barriers (major exclusion factor) by factoring inclusion of PWDs into a study’s impact score (subgroup recommendation) is likely to require creative adaptations to study protocols (TS principle: Creativity and Innovation).

## Recognizing PWDs as disparity population may reduce roadblocks to their inclusion in research

This section examines six major factors that contribute to PWDs being excluded from research: 1) funding structures, 2) eligibility and recruitment, 3) capacity and consent, 4) accessibility barriers in research, 5) PWD perspectives, and 6) researcher perspectives. We first summarize the literature for each (exclusionary) factor. Next, we identify how suggestions or commentary from NIH reports and/or roundtables (see below) about *implementing* the designation are in alignment with TS principles and its research agenda (see Table 1). The analysis draws from several resources:

**Table 1.** Main causes of PWD* exclusion form research, mapped to NIH subgroup recommendation and translational science principles

Exclusion Cause	NIH Subgroup Recommendation	TS Principles
Funding Structures	Establish Office of Disability Research (#2); Expand research on Disability Health and Health Disparities (#7a)	Address Unmet Needs
	Review NIH-funded workforce (#5)	Team Science
Eligibility and Recruitment	Increase PWD eligibility & enrollment in studies (#6); Track disability inclusion in impact scores (#7c; p.24)	Bold and Rigorous Approaches; Creativity & Innovation of methods
	Increase disability equity and access (#3); Disability members of workforce (#5) as means to increase inclusion (#7d)	Generalizable Solutions; Team Science
Capacity and Consent	Promote anti-ableism and anti bias (#4 & #8)	Bold and Rigorous Approaches; Generalizable Solutions; Cross-Discipline; Cross-Sector Partnerships
	Support inclusion of PWDs as research participants (#7d)	Creativity & Innovation of methods
Accessibility Barriers	Disability Equity and Access Coordinating Committee (#3)	Team Science; Cross-Sector Partnerships; Address Unmet Needs
	Expand inclusion of PWDs and PWD perspectives (#6); and address ableism in policies (#4) and workforce (#8)	Generalizable Solutions
	Support inclusion of PWDs as research participants (#7d)	Team Science; Address Unmet Needs; Generalizable Solutions
PWD Perspectives	Revise NIH mission statement (#1)	Address Unmet Needs
	Disability Equity and Access Coordinating Committee (#3); Include PWDs as research collaborators and on advisory bodies (#2, #6)	Generalizable Solutions; Cross-Sector Partnerships
	Review policy, culture & structure of NIH-funded workforce (#5); Address ableism in policies (#4) and workforce (#8)	Team Science
Research Perspectives	Fund research on PWD health / care disparities (#7b)	Bold and Rigorous Research Approaches

**Figure 2.**
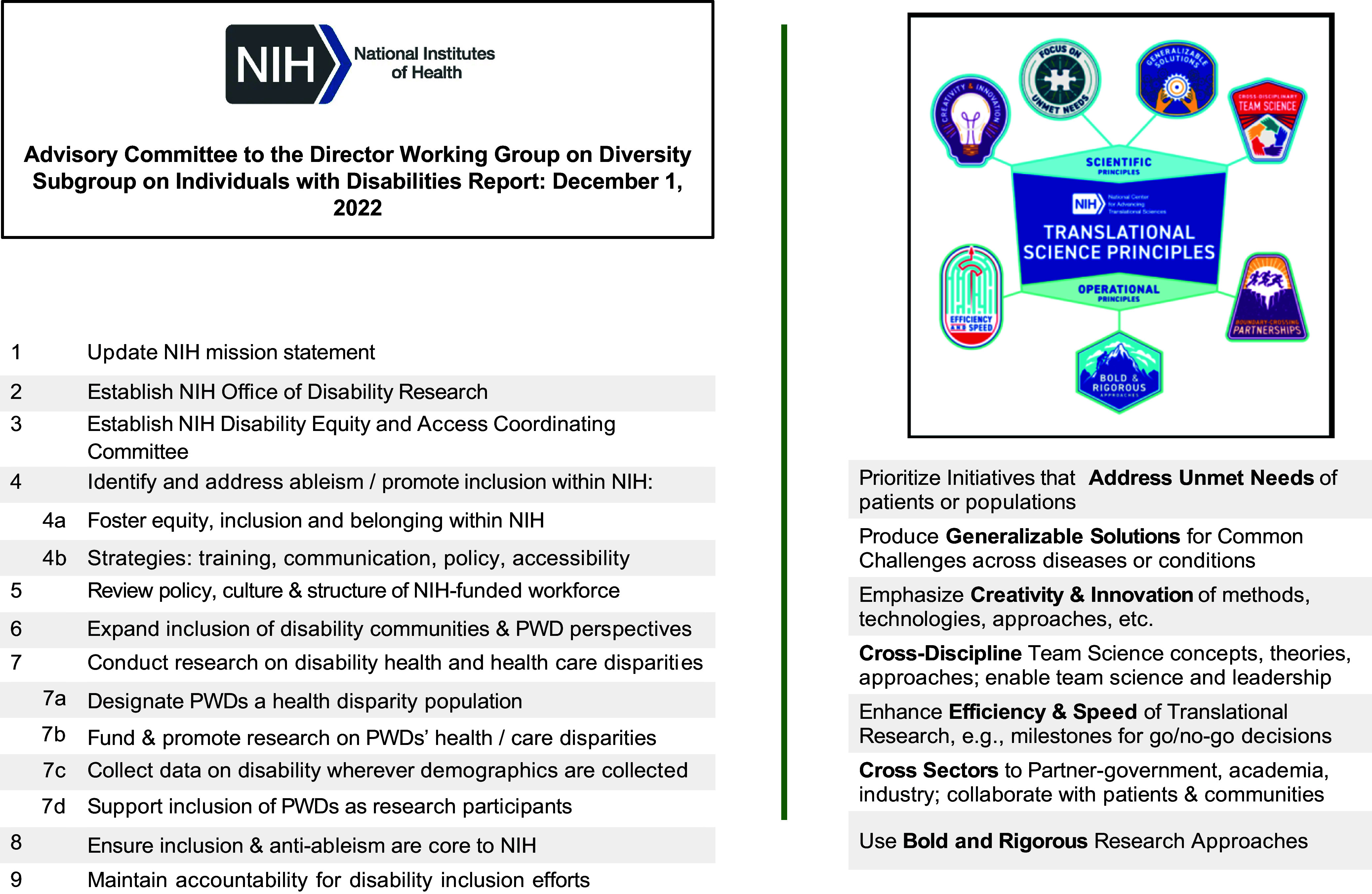
NIH Subgroup on disabilities recommendations (left) and translational science principles. Legend: PWD = people with disabilities.

* PWD = People with Disabilities.

- The 2022 NIH Subgroup on Individuals with Disabilities Report (“NIH Subgroup”), which originally formed to address disabilities in the NIH workforce, but then expanded to health disparities (Figure 2) [5].
- The 2023 *National Advisory Council to the National Institute of Minority Health and Health Disparities Working Group on Persons Living with Disabilities* (“NACMHD Report,” September 2023) [2] on PWD health disparities, research needs, and opportunities.
- The 2024 NIH Community Roundtables and Town Hall on Disability Research sought feedback on scientific opportunities and challenges, is organizational statement, how to encourage new disability researchers, and how to increase PWDs’ participation in research and clinical trials. Six roundtables were held (summaries available online); two each with persons with lived experience and advocacy organizations [10,11], clinicians and professional associations [12,13], and researchers [14,15].
- *Translational Science Principles* developed by the National Center for Advancing Translational Science (NCATS).
- *Translational Science Research Agenda* as promulgated by Austin in “Opportunities and Challenges in Translational Science” (2021) [16].

### Structure of research funding

PWDs – especially individuals with developmental disabilities – experience disparities across conditions (e.g., diabetes) not specific to their disability. Yet, much research funding is organized by condition [17]. To promote transparency, the NIH publicizes public funding for Research, Condition, and Disease Categories (RCDC). The RCDC lists more than 300 research area (e.g., nutrition), conditions (e.g., including those with high disability burden: depression, long COVID, migraine, and stroke) [18], and developmental disability categories (e.g., autism, Down Syndrome, intellectual, and developmental disabilities). Prior to June 2025 there had not been a discrete RCDC entitled “Disability Research.”

*TS Alignment:* The NIH Subgroup recommended establishing an Office of Disability Research and creating an RCDC category for disability research which “*differs from funding research on diseases, pathophysiological mechanisms, and health conditions that can cause disability* [emphasis added].” During its Town Hall, the NIH affirmed an agency-wide commitment to people and their disabilities [19]. Roundtable panelists urged inclusion of PWDs in all research [14], critiqued the siloing of research funding according to body function [12], and highlighted gaps in research on age-related chronic conditions (obesity, diabetes, CVD) among PWDs [12]. Creation of the new Disability Research RCDC is a positive step. Including PWDs in all research, not just that which is mechanistically or causally linked to disability would directly align with the TS principle of “Addressing Unmet Needs.”

### Eligibility and recruitment

Diverse samples enhance generalizability but may weaken internal validity. Unsurprisingly, broad and poorly justified eligibility criteria are cited as a reason for PWDs’ widespread exclusion from research. Exclusion can be *explicit*, e.g., “any other clinically significant abnormalities such as illiteracy or severe visual or hearing impairment,” and/or; *implicit* e.g., “any other reason the investigator deems exclusionary [20]. Psychiatric and cognitive conditions are the most commonly precluded disability categories [20,21]. When Camanni et. al. analyzed exclusion criteria in 1,000 trials, in most (96%) cases they deemed exclusions “relative,” i.e., more inclusive designs *could have* accommodated PWDs and still met the studies’ primary aims [21]. Overall, 35% of trials in that review explicitly excluded >= one disability With minor accommodations, another study found, >= 70% of persons with ID could have been included in the 300 studies analyzed [22].

Several strategies can reduce PWDs exclusion. Adopting standardized measures such as the Frailty Index, rather than relying solely on (potentially biased) clinical judgments of eligibility or chronological age is an example. Other strategies include alternative measures to assess primary constructs of interest [23], and developing universal design for testing methods [7]. In turn, larger samples with detailed baseline characteristics would then permit adjustment for PWD status and subgroup analyses [24].

*TS Alignment:* The NIH Subgroup recommended tracking disability inclusion metrics as part of NIH impact scores (p. 24) [5]. Roundtable panelists recommended that grant applicants be required to provide scientific justification for any exclusion involving disability [10,13] and that NIH inclusion enrollment reports explicitly emphasize planning and intentions to recruit PWDs [14]. Observing that the NIH Subgroup’s original main purpose was to expand PWDs’ participation in the research workforce, Roundtable panelists noted that including PWDs not only as participants but also as researchers would yield more finely tuned problem identification, framing, and external validity [13].

All people, regardless of current disability status, benefit from innovations originally designed for – and often by PWDs – such as speech-to-text and voice recognition technology [14]. To advance accessibility, cross-disciplinary collaboration (a core TS principle) can be used to develop and adapt assistive technologies. Such collaboration with expertise from engineering, artificial intelligence, can both make research more accessible and enhance the health and independence of PWDs [15].

### Capacity and informed consent

While the Belmont Report emphasizes both individual autonomy and protections for vulnerable populations, this has led to overly broad assumptions about decision-making capacity. Adults with DDs are frequently presumed to lack capacity to consent, despite evidence showing otherwise. For instance, among people with ID, over 75% have mild ID [25]. Moreover, co-occurring ID is relatively rare in many adult developmental disabilities – less than 1% for ADHD and about 30% for autism [26]. While clinical trials rarely explicitly exclude people with ID [22], implicit exclusions are common [27]. New strategies for assessing decision-making capacity including progressive quizzing and visual stories can make blanket exclusions unnecessary [28].

*TS Alignment:* The background reports call for robust training and education to prevent bias in recruitment that aligns with TS “Cross-Sector” and “Cross-Discipline” principles. Roundtable panelists proposed screening AI training data for negative disability stereotypes to prevent ableist bias, particularly when used as decision-making tools [10]. As another example, our “Disability as Diversity: Reducing Researcher Roadblocks” CTSA Element E project flagged pejorative language and lack of attunement to the variability of decisional capacity in our institution’s policy. Multiple stakeholders, including persons with I/DD and our Office of Human Research Affairs, collaborated to update the policy.

### Accessibility barriers in research practices

Accessibility encompasses three tactical layers: universal design, accommodations, and adaptive modifications [29]. *Universal design* refers to features that are accessible to all participants regardless of disability status, but are intentionally designed with disability needs in mind. Examples include REDCap’s built-in text-to-speech, setting adjustable fonts for eConsent as a default option [30], high-contrast text, audio captions, and ADA-compliant spaces. *Accommodations* address barriers when universal design isn’t feasible or successful, e.g., extra time, quiet rooms, sign language interpretation, and verbal consent. *Adaptive modifications* are more customized, e.g., switching from online to in-person testing (or vice-versa), modified exercises for wheelchair users, or including prosthetics in weight measurements.

*TS Alignment:* Beyond the subgroup’s initial focus on workforce accessibility, Roundtable panelists encouraged use of accessible materials (ASL, closed captions, plain language, speech-to-text) to in clinical trials [10], and funding for accessibility (physical, sensory, cognitive) formats [12]. These strategies align with TS aspirations to address unmet needs and ensure broad representation among participants in clinical trials. Additionally, implementing universal design throughout the research process – from conceptualization through accessible communication of results to affected communities, mitigates disease-agnostic roadblocks to NCATS core mission of “more treatments, to more people, more quickly.”

### Barriers from the perspective of PWDs

Mistrust, power differentials, and focus on cures versus quality of life contribute to PWDs’ skepticism of research. Without existing relationships built on trust, PWDs may feel “mined” by researchers [17]. A study of nearly 1300 adult PWDs (∼30% with a DD) identified researcher *lack of knowledge of their needs (40%)* or *stereotypes of PWDs (25%)* as barriers to participation. The most oft-cited facilitator was “knowing researchers are aware of the needs of people like me (65%),” while “knowing the study was respectful of PWDs” was cited by 58% [31]. For example, one of us (KB) led a Covid vaccine education project for persons with DD – a population with high Covid morbidity and mortality. Among parents of children with DD not planning to vaccinate their child, 80% cited lack of (known) inclusion of children with DD in the vaccine trials as a reason for their decision [32].

*TS Alignment:* Redressing these concerns requires change in the culture of science – away from a purely medical model of disability, towards one that encompasses the social model. Researcher panelists remarked that the terminology “reduce illness and disability” communicates that disability is mainly a problem that needs reducing rather than recognizing PWDs as a population with unmet needs [14,15]. They recommended asking questions that matter to the disability community, involving PWDs in developing funding opportunities, and allocating spaces on research teams for PWDs. Panelists with lived experience recommended that the statement recognize equal worth of PWDs, and funding research that maximizes their quality of life.

### Barriers from the perspective of researchers

While hundreds of studies document challenges in recruiting a wide range of underrepresented demographic groups, researchers’ attitudes about including PWDs remain understudied. A CTSA study revealed a gap between researchers’ theoretical support for heterogeneous samples (87%) versus prioritizing it in their own work (38%) [33]. A 2023 scoping review found 56 papers on recruiting PWDs to clinical trials, but only two addressed disabilities broadly rather than specific conditions. Of 22 papers examining researcher perspectives, none used quantitative methods; most were commentaries (15), case studies (6), or qualitative research (1) [24].

Researchers cite lack of resources as barriers to inclusion. In the above CTSA study of under represented populations, time, money, anxiety, and discomfort were viewed as “costs” to inclusivity [34]. Funding and time to support development of new methods and testing protocols, accessibility, and adaptive equipment are key [7,24,29]. Inclusive practices are often more burdensome for researchers whose work is already time and resource constrained [24]. Including persons with ID, for example, will require extra time as well as training regarding knowledge and skills for working with this population [35,36].

*TS Alignment:* Despite recognition of bias and ableism faced by PWDs and its perpetuation by *physicians*, there is less focus on the culture of science as it pertains to *researchers*. By contrast, researcher panelists were highly enthusiastic about including PWDs, but felt hampered by the lack of dedicated, sustained funding. More broadly, they acknowledged the pervasiveness of ableism in society and that a new conceptualization of disability could impact societal views. Establishing an Office of Disability Research could lead to meaningful change. Finally, they noted the minimal training researchers receive about disability inclusion [14,15].

## Disability as diversity: reducing researcher roadblocks (D2/R3): CTSA element E project

D2/R3 is our CTSA’s mixed-methods study of knowledge, attitudes, biases, and perceptions (KABP) related to including people with developmental disabilities (PWDDs) in research. It will develop and test tools to increase researcher motivation and capacity to mitigate perceived barriers to inclusion of PWDDs that will be applicable to other conditions. This section presents the specific aims of D2/R3, the project’s measures, and TS principles that the project most closely aligns with.

### D2/R3 description and aims

The specific aims of D2/R3 are to:

**Aim 1: Measure investigator and research team knowledge, attitudes, biases and perceptions (KABP).** 1a) *Engage PWDDs* to co-create vignettes for the KABP measures; 1b) *Develop and Conduct KABP Surveys* of investigators/team personnel (hereafter “researcher”) from the 10 sites; and 1c) *Develop Question Guide for Researcher-PWDD Conversation Circles* based on plain language summaries of survey results, emerging work to engage PWDDs in research, and PWDDs’ lived experience, for use in Aim 2.

**Aim 2: Co-design training on researcher-level factors that contribute to PWDDs’ under representation in research.** 2a) *Recruit* diversity of PWDDs who have chronic diseases associated with DD and investigators who study those diseases into virtual “*Bridging Research, Accurate Information and Dialogue*” dialogue circles that will 2b) *Co-Design* core content, themes, and messages for the training intervention, and 2c) Co-*Develop* eLearning modules informed by Transformative Learning Theory to test in Aim 3.

**Aim 3: Conduct randomized controlled trial of learning module** . We will recruit and randomize 200 researchers to an interactive on-line intervention, or an attention control module on research rigor and reproducibility.

### D2/R3 measures

To inform development of an educational intervention, D2/R3 will first measure each of the four KABP domains, as described below. Note, the Attitudes scale (described below) was the only pre-existing measure used in Aim 1 surveys; others were developed by the D2/R3 team.

- *Knowledge*: There are no empirical data on generalist clinicians’ or researchers’ knowledge of DD and health disparities of PWDD. Thus, Element E researchers elicited scenarios to incorporate into the Knowledge items, from PWDDs at Einstein-Montefiore’s UCEDDs program. We then developed a 10-item module covering topics such as the definition and prevalence of DD; ableist vs. non-ableist language, etc.
- *Attitudes (Primary Outcome):* D2/R3 used the WHO working group-developed *Attitudes to Disability Scale*. Its 16 items factor into 4 scales: Inclusion, Discrimination, Gains, and Prospects.
- *Bias*: Our team developed a novel Implicit Association Test (IAT) for DD through a multistep process. To identify stimuli terms for the IAT, we conducted a literature review, elicited input from PWDDs at our UCEDD, and conducted a survey to rate the valence and intuitiveness of 15 terms, from which we selected six terms.
- *Perceived Barriers*: We applied Prosci’s Perceived Barriers: We applied Prosci’s ADKAR® model of change management [37] to develop items that correspond to the 5 ADKAR outcomes needed for successful change.
  - ***A*** *wareness* of the need for change (e.g., recognition of PWDD health disparities [1 item]
  - ***D*** *esire* to enact and support the change (e.g., desire to enroll PWDDs in research studies [2 items]
  - ***K*** *nowledge* on how to change (e.g., use of universal design, plain language [4 items]
  - ***A*** *bility* to implement desired skills and behaviors (funds for extra time/resources [4 items])
  - ***R*** *einforcement* to sustain the change (e.g., NIH requiring a plan for disability inclusion [6 items])

The **K**nowledge, **A**bility, and **R**einforcement items drew upon Shariq et al.’s recent scoping review [24].

See supplementary file for Perception items, organized by 5 elements of Prosci’s ADKAR® framework.

### D2/R3 alignment with translational science

In this section we explicate D2/R3’s alignment with TS principles and priorities, as we did the designation of PWDs as a population with health disparities in Part II.

### Generalizable solutions for common challenges across diseases or conditions

Adults with DD are the focal population of D2/R3. This is because strategies that mitigate researchers’ perceptions of inclusion of adults with DD, will likely generalize to the 27% of US adults with any type of disability [1], whereas the reverse is less likely to be true. Adults with DD are a *subset* (precise prevalence unknown) of the nation’s 27% of adults with disabilities, and many of their DDs (e.g., autism, learning, and intellectual disabilities) are *non-visible.* Non-visible disabilities comprise 70%–80% of all disabilities; robust data finds stronger negative attitudes towards non-visible (vs. visible) disabilities [38]. Yet, the default image of disability is represented by the universal icon of a person using a wheelchair an image that does not encompass the non-visible characteristics of many DDs or chronic illnesses considered disabilities. And, because DDs are defined by childhood onset and lifelong impact – in contrast to general disabilities which often correlate with advancing age [39], DDs align with the CTSA lifespan approach.

### Initiatives that address meet unmet need of patients or populations

Compared to children, less is known about health care disparities of adults with DD re general health conditions (e.g., diabetes) not specific to their DD. In fact, there are no reliable data on the prevalence of adults with DDs in the USA. As rising numbers of children with autism and other DDs transition to adulthood, little is known about effective treatments for such conditions.

### Clinical trial participant recruitment, retention, and diversity

Austin’s translational science research agenda identifies this item as a “major rate-limiting translational problem…as a high-priority for innovation.” Historically, discussions about under representation in clinical trials have referred to racial and ethnic minorities [16]. The designation of PWDs as a population with health disparities is based on recognition of their under representation in research. As stated above, fewer than 5% of NIH research grants from 2018–2022 were related to disability, in contrast to the 27% of US adults with a disability (Figure 1).

### Cross-discipline / team science

Exemplifying cross-discipline research, D2/R3 brings together the only 10 US sites with a CTSA, Intellectual and Developmental Disabilities Research Center for basic/clinical research, and University Center of Excellence for Developmental Disabilities Education, Research, and Service (UCEDDs) program. Greater representation of PWDs in research must include investigators who design the study and eligibility criteria, study coordinators who manage logistics and accessibility, research assistants who recruit participants, etc. D2/R3 is collecting data from all team members.

## Discussion

Translational science aims to accelerate delivery of “more treatments for all people more quickly.” In this paper, we examined how applying TS principles to the designation of PWDs as a health disparities population can overcome roadblocks to PWDs’ inclusion in research. Several TS-related factors can facilitate overcoming these roadblocks: use of disability metrics, engagement of the PWD community, and potential “culture of science” effects in medical education and clinical care.

Metrics are key: “translation is only successful when it improves health of individuals and communities in tangible and measurable ways [16].” Notably, to “maximize the likelihood of improving attitudes and practices,” the NIH Subgroup wrote will require public monitoring – and disclosure – of its efforts [5]. Key recommendations from the NIH Subgroup and community roundtables included:

1. collecting data on disability wherever demographic data are captured in NIH systems.
2. including metrics of disability inclusion and accessibility as a component NIH grant impact scores
3. adding PWDs to inclusion policies for human subjects’ research
4. creating an RCDC for disability

In addition to metrics, the subgroup report and roundtables highlight the need to include the disability community as partners throughout the research process and to obtain their input on monitoring progress on recommended strategies. Previously, NCATS funded the creation of five online toolkits to engage PWDs and other under represented groups such as urban youth in research – but the development team lacked input from PWDs [40]. In contrast, the NIH Subgroup included a range of disability expertise, including at least two members who identify as PWDs (Drs. Iezzoni and Swenor).

Finally, to the extent that the culture of science evolves toward a hybrid medical-social model of disability, medical education, and clinical practice could be transformed. Currently, medical students receive little if any training on (developmental) disability, despite having keen interest [41], and recent AMA guidance that medical education infuse disability consciousness in health professions trainees [42]. There is no adult medical specialty analogous to Developmental-Behavioral Pediatrics. Instead, adult PWDs receive care from specialists such as Neurology (cognitive), Psychiatry (behavioral, mental health), and Physical Medicine & Rehabilitation (physical). By framing disability as an identity and not just a health condition, infusing disability content into undergraduate medical curriculum will likely improve all physicians’ cultural competency and awareness of accessibility needs.
